# Fascia and Muscle Stiffness in Soccer Athletes with and Without Previous Hamstring Injury

**DOI:** 10.3390/jfmk10010048

**Published:** 2025-01-28

**Authors:** Eleftherios Kellis, Afxentios Kekelekis, Eleni E. Drakonaki

**Affiliations:** 1 Laboratory of Neuromechanics, Department of Physical Education and Sport Sciences at Serres, Aristotle University of Thessaloniki, Agios Ioannis, 62110 Serres, Greece; afxentio@phed-sr.auth.gr; 2 Department of Anatomy, Medical School, University of Crete, 71110 Heraklion Crete, Greece; drakonakielena@gmail.com

**Keywords:** strain, hamstrings, soccer, injury, strength, elastrogram, modulus

## Abstract

**Background/Objectives:** Despite extensive efforts to reduce injuries to the hamstrings, the injury rate among athletes is increasing. The purpose of this study was to examine fascia and muscle stiffness differences between ten soccer players with a previous biceps femoris long head (BF) injury and thirteen controls. **Methods:** The shear-wave elastic (SWE) modulus and surface electromyography signal from the semitendinosus (ST) and BF were measured during passive and active knee flexion efforts from 0°, 45°, and 90° knee flexion angles. Anatomical cross-sectional area (CSA) and maximum isometric strength were also obtained. **Results:** Analysis of variance showed that the injured group showed significantly greater active (*p* < 0.05) but similar passive SWE modulus of BF and ST fascia and muscle than the uninjured group. Compared to the non-injured group, injured athletes had lower isometric strength and BF anatomical CSA (*p* < 0.05) but similar electromyographic activation amplitude (*p* > 0.05). **Conclusions:** The greater fascia stiffness during active submaximal contractions, in comparison to controls, might have an impact on hamstring function in soccer players with BF injuries who returned to play. Injured players may benefit from therapeutic interventions that aim to restore fascia and muscle tissue stiffness.

## 1. Introduction

Hamstring injuries are very common in sports, especially in soccer and athletics. Hamstring injuries can lead to a prolonged absence of an athlete from training or games and have a high recurrence rate [1]. Among the various risk factors, previous injury is one of the most widely accepted risk factors [2]. For example, a recent meta-analysis has shown that athletes with a previous hamstring strain have a 2.7-fold higher risk of suffering a new injury than uninjured individuals [2]. This raises the question of why hamstring strains require such a high recurrence rate.

Acute hamstring injuries with obvious structural damage are accompanied by edema and fluid accumulation, which gradually lead to the formation of a scar in the injured area [3,4], which affects the morphology and mechanical properties of the muscle-tendon unit [4,5,6]. By measuring the mechanical vibrations in response to external stimuli, some studies have found greater passive stiffness in the hamstring muscles of the injured compared to the non-injured leg [7,8] or compared to uninjured individuals [9]. However, using shear wave elastography (SWE), studies have found that athletes who have sustained a hamstring injury have similar [10,11] or lower [12] passive muscle stiffness scores during passive testing either between the legs or compared to uninjured individuals. The findings on stiffness during active contractions following a hamstring injury are also contradictory. Using SWE, some studies reported a lower biceps femoris long head (BF) active stiffness in the injured leg compared to the uninjured leg [13], while others found no differences [10]. In contrast, using the damped oscillation method, another study found greater overall hamstring stiffness in athletes with a previous hamstring injury than in non-injured athletes [14]. Furthermore, recent evidence indicated that athletes with a hamstring injury show greater SWE modulus of the lumbar musculature than controls [15]. The above shows that it is unclear whether athletes with a previous hamstring injury have different stiffness characteristics than non-injured athletes and, if so, what type of stiffness (active, passive, or both) is affected by the injury.

An injury to the hamstring can affect not only the mechanical properties of the muscle tissue but also those of the adjacent aponeurosis/fascia [5,16,17]. It is known that fascia tissues maintain connections with inter-muscular connective tissues, tendons, and ligaments [18], and they can offer an alternative pathway of force transmission to adjacent muscles [19]. A recent study has shown that during passive stretches and active contractions of the quadriceps muscles, there was a linear increase in stiffness of both muscle and fascia, highlighting the important role of fascia in optimizing muscles’ contractions [20]. Another study has found that athletes with a previous ankle sprain have thicker fascia tissue compared to uninjured athletes and suggested that this is due to lower muscle recruitment that occurs after injury [21]. This highlights the notion that injury impacts not only the muscle tissues but also the fascia structures, having an impact on the functioning of the whole myofascial unit.

Early studies have shown that injured muscles have less compliance of the proximal muscle-tendon junction due to scarring, resulting in greater elongation of muscle fibers during contraction [17] and a reduction in the aponeurosis strain, which is restored one year after the injury [22]. More recently, no difference was found in the size of the aponeurosis between the injured and non-injured leg or the uninjured participants [23]. Research has shown that during passive stretches and active contractions of the hamstrings, the SWE modulus of the fascia that surrounds the hamstrings (fascia latta) increases considerably, and it is greater for the ST compared to the semimembranosus fascia [24]. Further, changes in hamstring fascia modulus show a positive correlation with changes in spinal fascia tissue modulus [24,25]. Nevertheless, in those studies [24,25], only healthy individuals were examined, whilst the modulus of the fascia and muscle tissues was not compared between two synergist muscles, such as the BF and ST. To our knowledge, only Kawai et al. [11] studied fascia and muscle stiffness after hamstring injury and found that athletes with a previous injury had greater fascia but similar muscle BF SWE modulus in the injured hamstring compared to the uninjured hamstring. However, these results were only obtained in passive tests, while the fasciae of the other synergistic hamstrings were not examined. There are suggestions, however, that injury may affect the function of other synergists [26,27] under both passive and active conditions [6,28]. If an injury of the hamstring influences both muscle and fascia function, then this should indicate that therapeutic strategies targeting both muscle and fascia should be appropriate for managing individuals with a recent hamstring injury.

The aim of this study was to investigate the differences in semitendinosus (ST) and BF muscle and fascia stiffness in athletes with a previous hamstring injury and control subjects. It was hypothesized that athletes with a previous injury would have higher overall stiffness compared to controls. Furthermore, the group differences in stiffness would be more pronounced in fascia than muscle tissue, and finally, individuals with a previous hamstring strain would have greater BF but similar ST stiffness compared to uninjured individuals.

## 2. Materials and Methods

### 2.1. Design

This was a cross-sectional study, and it was part of a large project that compared the muscle and fascia stiffness of athletes with a previous hamstring strain with non-injured athletes. Based on preliminary statistical testing using G*Power software (Version 3.1.9.4, Universität Düsseldorf), a minimum of 15 participants were estimated as necessary to detect group differences with a medium effect size of 0.31, with a statistical power of 80% and type 1 error of 0.05. Eight Greek League semi-professional clubs were contacted to recruit players who had sustained a hamstring injury within the last year and successfully returned to play, as well as players who had no other musculoskeletal injuries, pain, or other pathological conditions that led to absence from training or play during the last year. A total of 151 players responded to the first call. Of these, 121 players took part in a screening by one of the researchers, who is a qualified doctor and radiologist.

Athletes who had sustained a hamstring injury answered a questionnaire to determine the mechanisms and conditions of the injury. The hamstring injury was defined as acute pain in the posterior thigh that caused the player to miss either training or matches [29]. Twenty-two athletes with a grade II BF strain confirmed by MRI or US findings were selected for the hamstring injury group. Of these, 12 were excluded (4 had concurrent injuries, 5 had injuries to another hamstring muscle, 2 had a mild injury with minimum absence from play, and one failed to participate in the measurement sessions). The demographic characteristics of the injured group were measured (N = 10; age: 29.0 ± 6.61 years; mass 78.9 ± 8.02 kg; height 181 ± 9.71 cm). The time between testing and injury was 4.4 ± 3.2 months; their time lost due to injury was 34.8 ± 3.9 days, while for 4 athletes, this was a recurrent injury. This information was then used to form the uninjured group by matching the injured and uninjured players based on their chronological age. Subsequently, 13 uninjured players formed the uninjured group (age: 27.3 ± 4.7 years; mass 71.0 ± 4.5 kg; height 174.1 ± 4.65 cm). The participants gave their written consent, and the protocol was approved by the Institutional Ethics Committee of the Department of Physical Education and Sport Science at Serres, Aristotle University of Thessaloniki (ERC/018/2022) in accordance with the Declaration of Helsinki.

### 2.2. Procedures

All tests were carried out with the subject in a prone position, the hips in a neutral position, and the hands next to the body. A wide elastic strap was used to maintain the pelvic position. The maximum isometric strength (MVC) values of the knee flexors of the right leg from 0° (=full extension), 45°, and 90° knee flexion angles were evaluated. Before the test, the participant completed a warm-up training with static stretching of the hamstring and several submaximal static contractions. In each knee flexion angle, participants were asked to exert maximum knee flexion effort against a hand-held dynamometer (K-Force muscle controller, sampling rate 75 Hz, Kinvent, Montpellier, France). The force sensor was positioned just above the lateral malleolus. The distance between the dynamometer placement and the lateral epicondyle was measured and used to calculate the torque exerted around the knee. In each joint position, the participant performed 3 MVC efforts of 5 s each. The maximum torque was taken as the MVC value.

The SWE and EMG measurements were then evaluated under two conditions: passive and active. In the passive condition, measurements were taken at rest while the knee was held at knee flexion angles of 0, 45, and 90° for 5 s in randomized order. In the active condition, the participant performed submaximal contractions at each angle. The target strength level was set at 60% MVC. The participant had about 2 s to gradually reach this level and then hold it for about 5 s. Measurements were taken separately for each muscle in random order with a ten-minute rest period in between. Three trials per condition were recorded.

### 2.3. SWE Measurements

US measurements were performed with the LOGIQ E9 ultrasound system (version R5, General Electric, Chicago, IL, USA) using an ML6-15 (4–15 MHz) linear array transducer and a 9 L (2–8 MHz) 2D linear transducer for B-mode and elastography measurements, respectively. The software integrated into the system automatically calculates Young’s modulus of elasticity in kilopascals (kPa) using the equation E = ρ·V^2^, where E is Young’s modulus of elasticity and ρ is the tissue density (assumed to be 1 g/cm^3^), and V is the velocity of the shear waves [30].

**Figure 1:**
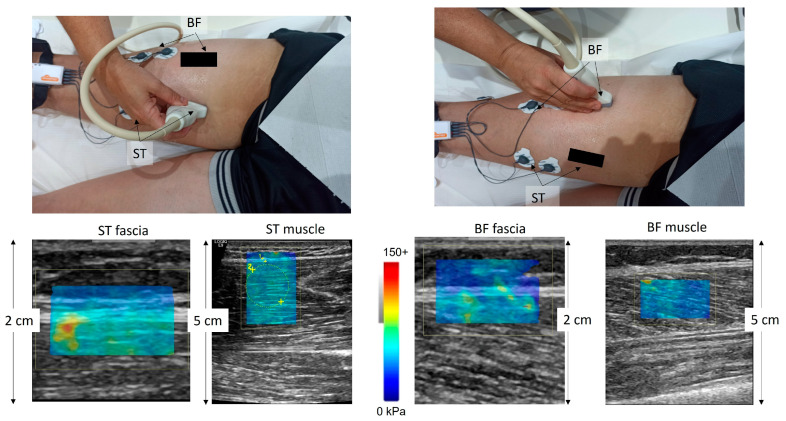
The experimental set-up. The ultrasound probe was placed on the semitendinosus (ST) and the biceps femoris long head (BF) at 40% of the distance from the ischial tuberosity to the distal ends. Elastograms were superimposed on B-mode images as color-coded boxes. For fascia tissue analysis, the images were zoomed so that the fascia tissue was enlarged and separated from the irrelevant tissues. Circles were then drawn inside the fascia or the muscle tissue, and the shear modulus values were calculated. The color scale was extracted from the software and is enlarged so that the measurement scale is easily visible.

The exact locations were first identified on the tested side of the body with B-mode ultrasound and marked on the skin (Figure 1). The probe was placed approximately 40% of the distance between the medial condyle (for the ST) or fibula (for the BF) and the ischial tuberosity. After identifying the area of interest in the axial plane and applying ultrasound gel, the probe was moved longitudinally and parallel to the muscle fibers to perform SWE measurements.

Using the US system default standard musculoskeletal setting (Figure 1), modulus measurements in kilopascal (KpA) were presented in a rectangular (approximately 4 cm × 3 cm) color-coded box (elastogram). Manually selected circular regions of interest (ROIs) were placed on the fascia and the muscle to obtain the SWE modulus. For ST, ROIs were selected from the muscle/fascia area, which is located distally to the tendinous inscription. Once the US image was obtained, two types of measures were obtained. First, the ROIs included only the hyperechoic fascia layer, excluding subcutaneous fat and muscle. The outcome variables were ST fascia (STF) modulus and BF fascia (BFF) modulus. Second, in the same image, ROIs were obtained to cover most of the respective muscle area available in the elastogram and exclude the fascia planes from each ROI. The outcome variables from this analysis were ST muscle modulus and BF muscle modulus. All measurements were determined by a radiologist with 18 years of experience. The mean SWE modulus values in each ROI and the mean values of the three ROIs acquired in each trial were calculated. The reliability of SWE values was checked in 5 individuals who were examined by the same tester in two separate sessions spaced at least 1 day apart. The intraclass correlation coefficient (ICC version 3.1) values were 0.91 (STF), 0.89 (ST), 0.88 (BFF), and 0.94 (BF) for passive tests and 0.87 (STF), 0.86 (ST), 0.89 (BFF) and 0.91 (BF) for submaximal contractions, indicating high reliability.

### 2.4. EMG Recording and Analysis

Bipolar surface electrodes with a 1cm distance in between were used to record the EMG signal using two wireless Shimmer3 EMG devices (Shimmer Research Ltd., Dublin, Ireland). The placement of the electrodes followed the guidelines of the SENIAM project, slightly modified so that US probes and EMG signals are recorded simultaneously [31]. After shaving and cleaning the skin with alcohol wipes, the electrodes were placed 60% of the distance between the proximal and distal ends of each muscle. A common ground electrode was applied on the epicondyle of the untested leg. The signal was sampled using a 24-bit analog-to-digital converter with a sampling rate of 1024 Hz and a gain of 1000 (common mode rejection ratio of 120 Db, input impedance = 100 MΩ). It was then filtered using a band-pass filter (between 15 Hz and 450 Hz), and the full wave was rectified. The root mean square (RMS) was calculated with a step of 50ms. Following EMG data collection, the maximum RMS value produced during the isometric MVC of each muscle was taken as a reference measurement. Subsequently, the RMS during each testing condition was normalized to the maximum value recorded during the MVC tests.

The anatomical cross-sectional area (CSA) of ST and SM (in cm^2^) was measured in 8 injured and 12 controls. With the participant in the prone position, a transverse panoramic US scan was performed at 40% of the line between the greater trochanter to the outer femoral condyle. The investigator moved the probe slowly and carefully, following a path drawn on the skin from the medial to the lateral side of the hamstrings. Three panoramic images were obtained. The contours of the ST and BF were manually digitized on the recorded panoramic US image using an image-based software (MicroDicom Viewer, v2023.3 MicroDicom Ltd., Sofia, Bulgaria). The ROI could be selected with as much of the muscle as possible without including any surrounding bone or fascia.

### 2.5. Statistical Analyses

Statistical analysis was performed using the Statistical Package for social sciences (v 29.0. IBM Corp, Armonk, NY, USA). Normal distribution was confirmed using Shapiro–Wilk tests. Hence, differences in SWE modulus between joint angles (3 angles), groups (control, injured), tissues (BF, ST, BFF, and STF), and condition (passive, active) were examined using a four-way analysis of variance (ANOVA). A separate four-way ANOVA was also used to examine the effects of all independent variables on normalized RMS signals. Differences in MVC torque and CSA were examined using ANOVA or independent *t*-tests, respectively. Effect sizes were also calculated using the partial eta squared (η^2^) or d values [32]. If significant, post hoc Tukey tests were applied to examine significant differences between pairs of means. The alpha level of significance was set at a = 0.05.

## 3. Results

SWE modulus values for each condition are presented in Table 1. The four-way (group × condition × tissue × angle) interaction effect was not statistically significant (*p* > 0.05). Three-way interaction effects not involving the group or non-significant interaction effects involving the group are not reported. The results showed a statistically significant main effect for the group (F_1,21_ = 4.91, *p* = 0.038, η^2^ = 0.586). SWE modulus (averaged across all testing conditions) was greater in the injured compared to the non-injured group. Statistically significant main effects for tissue (F_3,63_ = 29.37, *p* = 0.0001, η^2^ = 0.978), joint angle (F_2,42_ = 5.18, *p* = 0.010, η^2^ = 0.801), condition (F_1,21_ = 586.66, *p* = 0.0001, η^2^ = 0.965) were found. Post hoc analysis showed that the average SWE modulus was greater for fascia tissues compared to their corresponding muscle, and it was greater for ST compared to BF (*p* > 0.05). Finally, the SWE modulus (averaged for all testing conditions) was greater at 0° compared to 45° and 90° values (*p* > 0.05).

**Figure 2:**
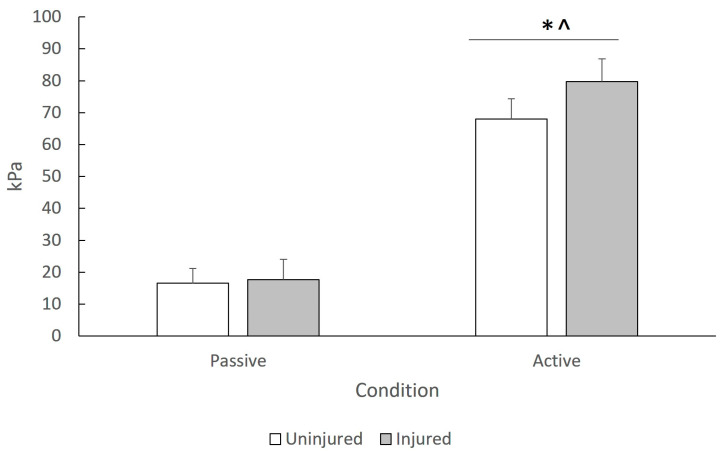
Mean SWE modulus (average value of all tissues and angular positions) during passive and active conditions. Error bars indicate standard deviations (* indicates statistically significant difference between groups, *p* < 0.05; ^ indicates statistically significant difference compared to passive tests, *p* < 0.05).

From the interaction effects, the ANOVA indicated a statistically significant group X condition interaction (F_1,21_ = 5.11, *p* = 0.018, η^2^ = 0.197). To better illustrate this interaction effect, Figure 2 presents the SWE modulus values (averaged across tissues and joint angles) of each group in passive and active conditions. Post hoc Tukey tests showed that the injured group showed significantly greater SWE modulus (averaged for all tissues and angles) than the uninjured group during active contraction only (*p* < 0.05).

Table 2 presents the EMG results. The four-way interaction effect was not statistically significant (*p* > 0.05). Similarly, none of the interaction effects involving the group were statistically significant (*p* > 0.05). All remaining interaction effects that did not involve the group are not reported. With regard to the main effects, the ANOVA showed no differences between the two groups in any of the testing conditions (*p* > 0.05). The EMG (averaged for all muscles and testing conditions) was greater at 0° and 45° compared to 90° (F_2,42_ = 16.07, *p* = 0.0001, η^2^ = 0.434) as well as during contraction compared to resting values (F_1,21_ = 289.28, *p* = 0.0001, η^2^ = 0.932) (*p* < 0.05). No differences in normalized EMG between muscles were observed (*p* > 0.05).

Table 3 presents the group results of MVC torque and CSA. MVC torque was greater in the injured compared to the uninjured group (F_1,21_ = 9.46, *p* = 0.006, η^2^ = 0.835), and it was greater at 45° compared to 90° angle (F_2,42_ = 3.52, *p* = 0.039, η^2^ = 0.622). In addition, a significantly lower BF CSA in the injured compared to the non-injured group (t_18_ = 2.45, *p* = 0.012, d = 1.12) was found, while there was no group difference in ST CSA.

## 4. Discussion

The main findings of this study were that soccer players with a previous hamstring injury had higher overall hamstring stiffness compared to controls. The differences were evident during active but not passive tests. These differences were similar for fascia and muscle as well as for BF and ST. Finally, injured athletes had lower knee flexion strength and BF CSA but similar EMG amplitude compared to uninjured individuals.

The findings of this study partly confirmed the first hypothesis, as previously injured athletes had similar passive but greater active fascia and muscle stiffness than controls (Table 1). These results agree with previous SWE findings on passive muscle stiffness [10,11], but they contrast with Freitas et al. [13], who reported that injured had lower active SWE stiffness than controls, while Freitas et al. [10] found no influence of previous injury on active SWE stiffness. In contrast, the present results agree with research studies that implemented mechanical oscillations to quantify stiffness and reported greater passive [7,8] or active hamstring stiffness either between limbs or between injured and non-injured athletes [9,14]. Furthermore, the present findings partly agree with a previous study [11], which reported that athletes with a hamstring injury had a higher passive fascial SWE modulus but a similar muscle modulus in their injured compared to uninjured leg. These conflicting results may be due to methodological differences. For example, in the present study, active stiffness was measured at 60% MVC, which is much higher than the intensity used in previous studies (20% MVC) [10,13]. The mechanisms of stiffness and force development depend on the intensity of exertion [33], and this may be altered following hamstring injury. In addition, the methods used to diagnose injuries vary between studies, from self-report [10] to MRI/US-based evaluation ([13] and the present study). Furthermore, in the present study, we only included individuals with BF strains that occurred no more than 12 months after injury (mean 4.4 months), whereas, in previous studies, individuals with injuries to each hamstring occurred on average 1.2 to 1.5 years before testing [10,13]. Even though the athletes in all studies returned to play, it is quite possible that the tissue recovered further in the athletes who sustained an injury 1.5 years ago than in the individuals who were injured 6 months ago [34]. This is reinforced by the lower MVC capacity observed in this study (Table 3), whereas no such deficits were observed in previous studies [10,13].

Due to the cross-sectional nature of this study, it is unknown whether the greater active SWE modulus that was observed in the injured limbs existed prior to injury or was due to injury. It seems logical, however, to consider previous evidence according to which scarring and the healing process following a hamstring injury can lead to an increase in stiffness in the injury region and adjacent tissues that may persist beyond the time of return to play if it resolves at all [34]. Scar development influences the hydration of the fascia [35], causing alterations in fascia and muscle gliding [36], which in turn can lead to an increase in its stiffness [4,11]. An alternative possibility is that the elevated hamstring stiffness in injured players existed prior to their injury, in which case mechanisms that are related to the physical conditioning of players lead to an increase in stiffness (lower strength, lower muscle size, or joint flexibility) may be activated [14].

Group differences in SWE modulus were evident during contractions and not during passive tests (Figure 2), which agrees with some studies [10] but not with others [13]. To the best of our knowledge, no previous study examined facia/aponeurosis stiffness in athletes with a hamstring injury during active contractions. One hypothesis might be that as the healing process continues, the stiffness of the involved tissue gradually recovers, thus having less influence on the extracellular matrix and myofilament elasticity and, hence, less passive SWE modulus of the injured myofascial tissue. In contrast, alterations in actomyosin cross-bridge formation and myofascial tissue could increase active SWE modulus [37]. Contraction of the muscle-tendon unit requires lengthening of the tendon and shortening of the muscle fibers. Hence, a decrease in tendon/fascial compliance after injury may continue to increase active stiffness when muscles actively contract [17]. Interestingly, players with injuries had lower strength levels than uninjured players (Table 3). This stiffness increase and force loss have been observed after injury [34] as well as after exercise-induced muscle damage protocols, and they were seen as a protective response against subsequent exercise-induced muscle damage [17,38].

The third hypothesis of the study could not be confirmed, as the injured group showed greater stiffness not only in the BF but also in the ST (Table 1). Since ST and BF share a common proximal tendon, injury to the fascial muscle tissue in BF may influence the immediately adjacent ST. Experiments in animal muscles have shown that scar tissue formation and fascial remodeling can create a stiff pathway of force transmission to the adjacent synergistic muscles [39,40]. It should be mentioned, however, that Freitas et al. [13] found that injured limbs showed different BF SWE modulus than non-injured limbs, while there was no difference in ST SWE modulus. As this study tested athletes in a shorter post-injury period than other studies [10,13], it is possible that the stiffness of the ST continues to be influenced by the injury. Further research is needed to investigate the changes in the mechanical properties of the myofascial tissue adjacent to the injured tissue to confirm these suggestions.

Although injured athletes had higher active stiffness (Figure 2), their strength and CSA (Table 3) were lower than control subjects. Other studies reported no differences in isometric strength after injury [10,13], which may (partly) explain why they reported different results on active stiffness compared to the present study. Systematic reviews have reported that there is limited evidence that isometric strength [41], CSA [6], or EMG [27] differ in injured compared to uninjured limbs. This reflects the small number of studies available [6] and the considerable variability of study participants and methods used. In the specific sample of injured athletes examined in this study, the lower strength values can be attributed to atrophy of the BF (Table 3) rather than lower neuromuscular activation (Table 2). This is in line with reports which suggested that injury can cause an increase in stiffness of the near myofascial tissues whilst there is a reduction in strength capacity [34,42].

This study had some limitations. Even though we screened more than 100 soccer athletes, about 20% had hamstring injuries; of these, about 80% had a BF grade II injury, which was diagnosed using an imaging method. This resulted in a relatively homogenous injured group of athletes (same sport, similar time since injury, and same muscle injured), which was relatively small in size. In addition, the mechanism of injury was unclear, and, further, the rehabilitation protocol followed by injured athletes was not controlled; therefore, its impact on neuromuscular status is unknown. Finally, SWE modulus results are specific to the probe location; it is possible that measurement in a different location yields different results [30].

The results may have some implications for the management of injured players and the prevention of recurrent injuries. The increased fascia SWE modulus in athletes with previous strain adds to previous studies [5,23], which emphasize the important role of aponeurotic tissue morphology and its mechanical properties as an injury risk factor or as a factor that influences movement of athletes with a history of injury. Irrespective of whether these changes pre-existed or occurred after injury, the increased fascial stiffness in the injured limbs indicates a different interaction of muscle and tendon/aponeurotic tissues during active contractions (compared to non-injured players), potentially decreasing the force generation capacity of the muscle-tendon unit [17,38]. Based on the present findings, athletes who return to play after a BF grade II injury may be in need of management of their increased stiffness in the injured region at least for the first 6 months after injury. Strategies may involve therapeutic interventions aiming to restore not only muscle fiber functionality but also to assist in the recovery of fascial tissue morphology and function to pre-injury levels. For example, studies have shown that hamstring stiffness decreases following trunk stability programs [43] or eccentric exercise at longer lengths [44], while a consensus statement suggested that alteration in fascia stiffness can result from manual therapies or foam-rolling techniques, although their efficacy remains to be validated [45]. Future research could examine whether such greater active stiffness from the fascia and muscle has implications for injury and re-injury risk.

## 5. Conclusions

Soccer players with previous hamstring injuries in the last year had greater active SWE modulus of both fascia and muscle and both ST and BF than uninjured athletes. Within this study’s limitations, the greater fascia stiffness during active submaximal contractions, in comparison to controls, might have an impact on hamstring function in soccer players with BF injuries who returned to play. Further, therapeutic interventions following hamstring injury may aim to restore not only muscle but also fascia function.

## Figures and Tables

**Table 1:** Mean (±SD) shear-wave modulus of semitendinous and biceps femoris fascia and muscle in injured (N = 10) and uninjured (N = 13) athletes. The significance of the main effects for condition, group, angle and tissues is also presented.

Knee Angle (°)	STF		ST	
	Uninjured	Injured	Uninjured	Injured
Passive				
0	20.9 ± 6.6	24.8 ± 15.3	18.2 ± 4.6	16.7 ± 4.1
45	13.5 ± 3.8	13.7 ± 9.1	12.5 ± 3.7	12.6 ± 5.9
90	11.3 ± 2.2	11.2 ± 3.8	10.8 ± 2.6	12.2 ± 2.6
Active				
0	85.5 ± 34.9	107.3 ± 31.3	69.0 ± 15.8	77.3 ± 17.6
45	78.9 ± 29.9	108.5 ± 38.7	66.1 ± 14.3	80.2 ± 13.6
90	94.4 ± 35.7	92.1 ± 19.5	69.5 ± 11.2	81.4 ± 15.8
	BFF		BF	
Passive				
0	29.9 ± 20.3	37.3 ± 22.1	19.6 ± 9.7	18.4 ± 8.8
45	18.1 ± 8.2	19.1 ± 8.8	14.5 ± 6.4	19.6 ± 8.3
90	13.4 ± 7.5	13.8 ± 4.2	15.7 ± 8.3	12.1 ± 2.5
Active				
0	68.5 ± 20.8	71.8 ± 9.1	53.1 ± 10.4	65.8 ± 25.0
45	64.3 ± 19.8	74.7 ± 20.3	54.0 ± 14.8	65.9 ± 10.2
90	59.9 ± 16.2	68.6 ± 15.3	52.9 ± 11.6	63.1 ± 18.4
Main effects (*p* < 0.05)				
Condition		Active > passive		
Group		Injured > Uninjured		
Angle		0° > 45°, 90°		
Tissue				
Fascia vs. muscle		STF > ST, BFF > BF;		
Between-muscles		STF < BFF, ST < BF		

STF = semitendinous fascia, ST = semitendinosus muscle, BFF = biceps femoris long head fascia, BF = biceps femoris long head muscle.

**Table 2:** Mean (±SD) normalized electromyography amplitude (percentage of maximum voluntary contraction) of the biceps femoris long head and semitendinosus in injured (N = 10) and uninjured (N = 13) athletes. Significance for the main effects of condition, group, angle and muscles are also presented.

Knee Angle (°)	ST		BF	
	Uninjured	Injured	Uninjured	Injured
Passive				
0	10.3 ± 5.2	9.7 ± 3.7	9.6 ± 4.0	11.0 ± 6.4
45	11.4 ± 6.6	10.0 ± 4.6	12.6 ± 5.8	13.0 ± 7.2
90	8.4 ± 6.3	9.5 ± 4.7	5.6 ± 3.5	6.5 ± 3.2
Active				
0	52.2 ± 11.9	66.9 ± 21.9	54.0 ± 14.0	53.3 ± 6.8
45	47.4 ± 13.5	64.8 ± 23.3	51.9 ± 12.5	56.1 ± 12.1
90	37.5 ± 10.9	58.0 ± 28.7	39.2 ± 18.4	37.2 ± 13.3
Main effects (*p* < 0.05)				
Group		NS		
Angle		90° < 0°, 45°		
Muscles		NS		
Condition		Active > Passive		

ST = semitendinosus muscle, BF = biceps femoris long head muscle, NS = not significant.

**Table 3:** Maximum isometric strength (MVC) at different knee flexion angles and anatomical cross-sectional area (CSA) of the biceps femoris long head and semitendinosus in the injured and uninjured group.

	Injured	Uninjured
MVC (Nm)		
Angle (°)	N = 10	N = 13
0	93.4 ± 17.5 *	122.8 ± 26.1
45	99.6 ± 18.8 *^,^^	131.1 ± 27.5 ^
90	94.0 ± 14.0 *	118.1 ± 32.3
CSA (cm^2^)	Injured	Uninjured
	N = 8	N = 12
BF	13.2 ± 1.2 *	14.6 ± 1.2
ST	12.8 ± 2.3	12.6 ± 2.2

* = Significantly different compared to uninjured group, *p* < 0.05. ^ = Significantly different compared to 90°, *p* < 0.05. ST = semitendinosus muscle, BF = biceps femoris long head muscle.

## Data Availability

Data are available at Mendeley Data, V2, https://doi.org/10.17632/sphbcgbtz7.1; date accessed: 25 January 2025.
